# Supplementary material for: Massive extended streamers feed high-mass young stars
Source: Sci Adv. 2025 Aug 20;11(34):eadw4512. doi: 10.1126/sciadv.adw4512 (PMC12366688; doi:10.1126/sciadv.adw4512)
Supplement: Supplementary file 1 — Figs. S1 to S4 [file sciadv.adw4512_sm.pdf]

Science Advances
AAAS

Supplementary Materials for

Massive extended streamers feed high-mass young stars

Fernando A. Olguin *et al.*

Corresponding author: Fernando A. Olguin, f.olguin@yukawa.kyoto-u.ac.jp

Sci. Adv. **11**, eadw4512 (2025)
DOI: 10.1126/sciadv.adw4512

This PDF file includes:

Figs. S1 to S4

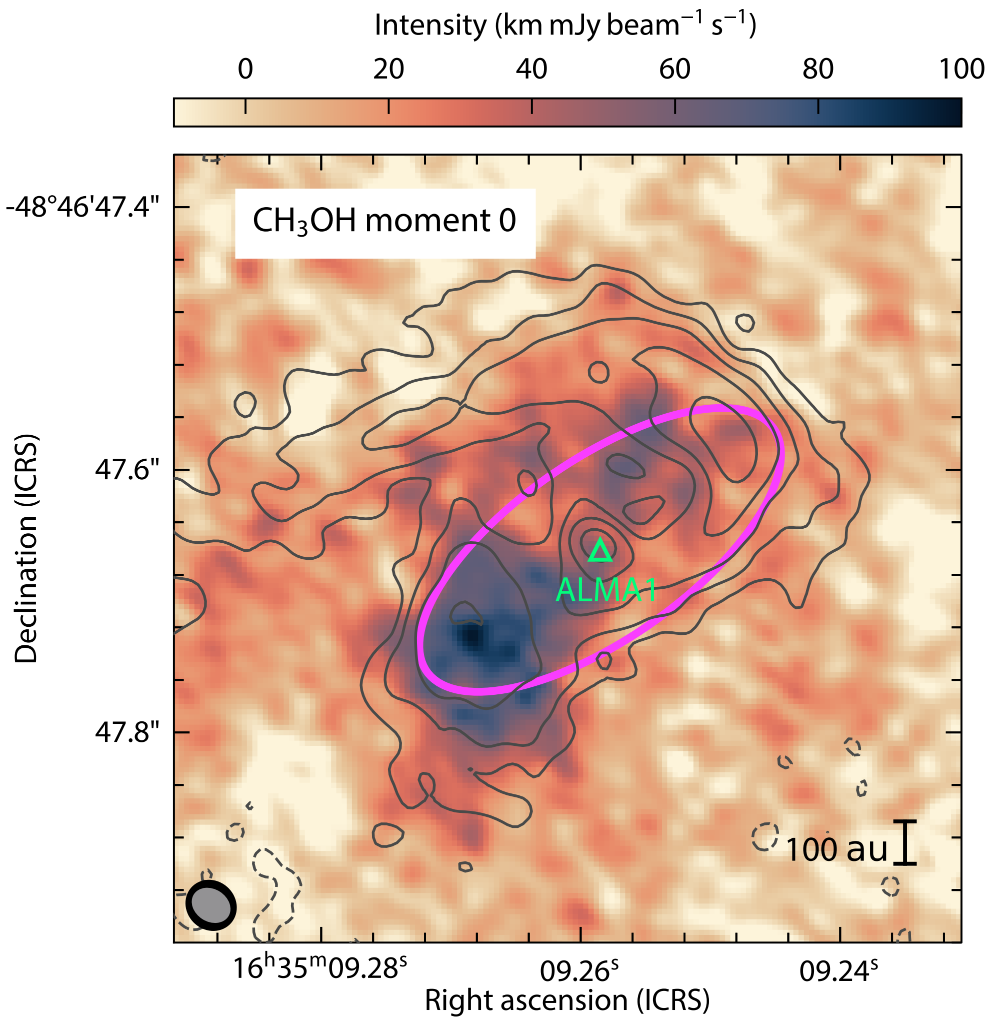

Figure S1: **Zeroth order moment of CH_3OH in color scale with continuum in gray contours.** The contour levels are the same as in Fig. 1. The pink ellipse shows the projected size of a disk of radius 500 au. The synthesized beam are shown in the bottom left corner for the zeroth order moment map (black) and continuum (gray).

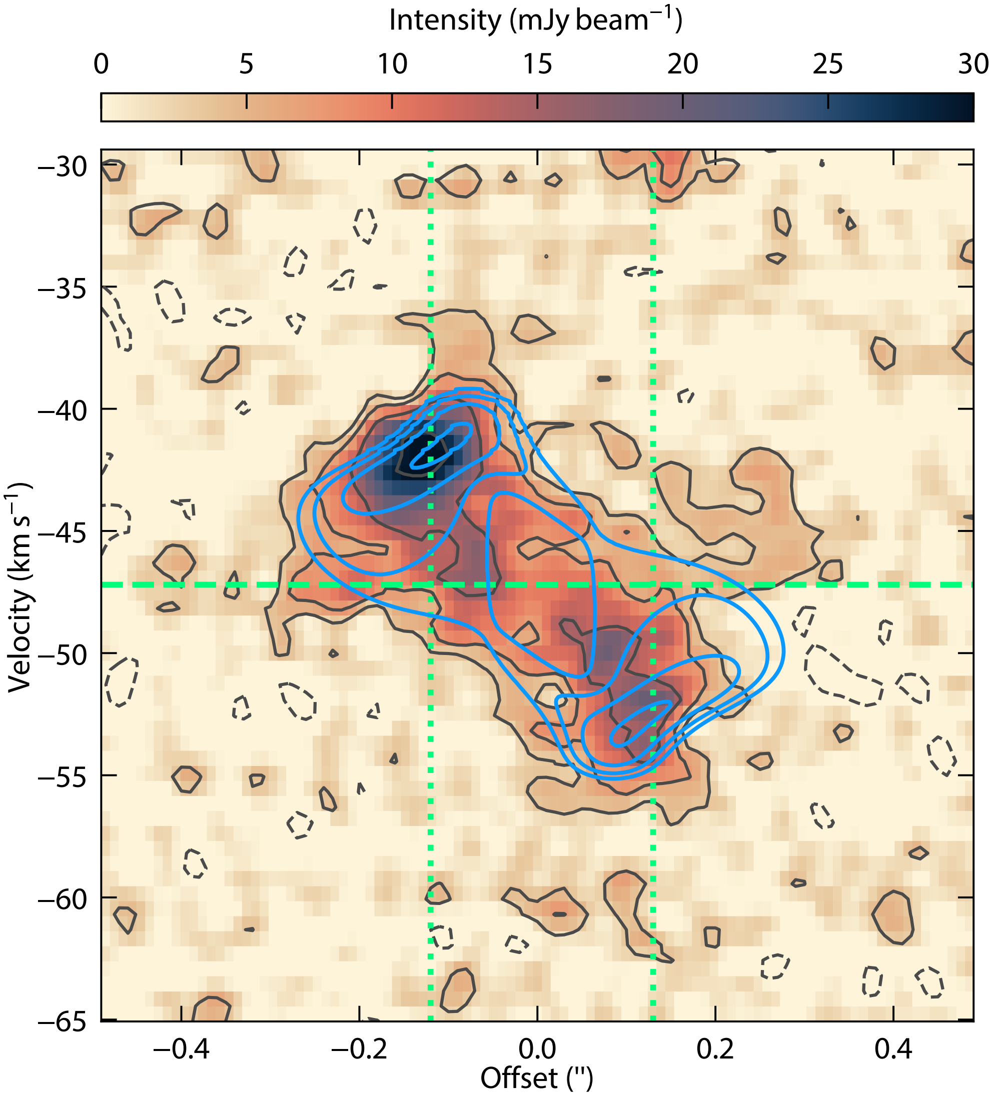

Figure S2: **Observed position-velocity diagram of CH_3OH along the rotation direction (P.A. = −55°) from (*28*) in color scale and gray contours, and updated IRE model with a centrifugal barrier of 250 au in blue contours.** Gray contour levels are −6, −3, 3, 6, 12 and $24 \times \sigma$ with σ = 1.2 mJy beam^{-1}, while blue contours are at the same levels after re-scaling to match the CH_3OH peak emission. The horizontal green dashed line corresponds to the systemic velocity (−47.2 km s^{-1}), and the vertical dotted lines correspond to the offsets of the continuum peaks.

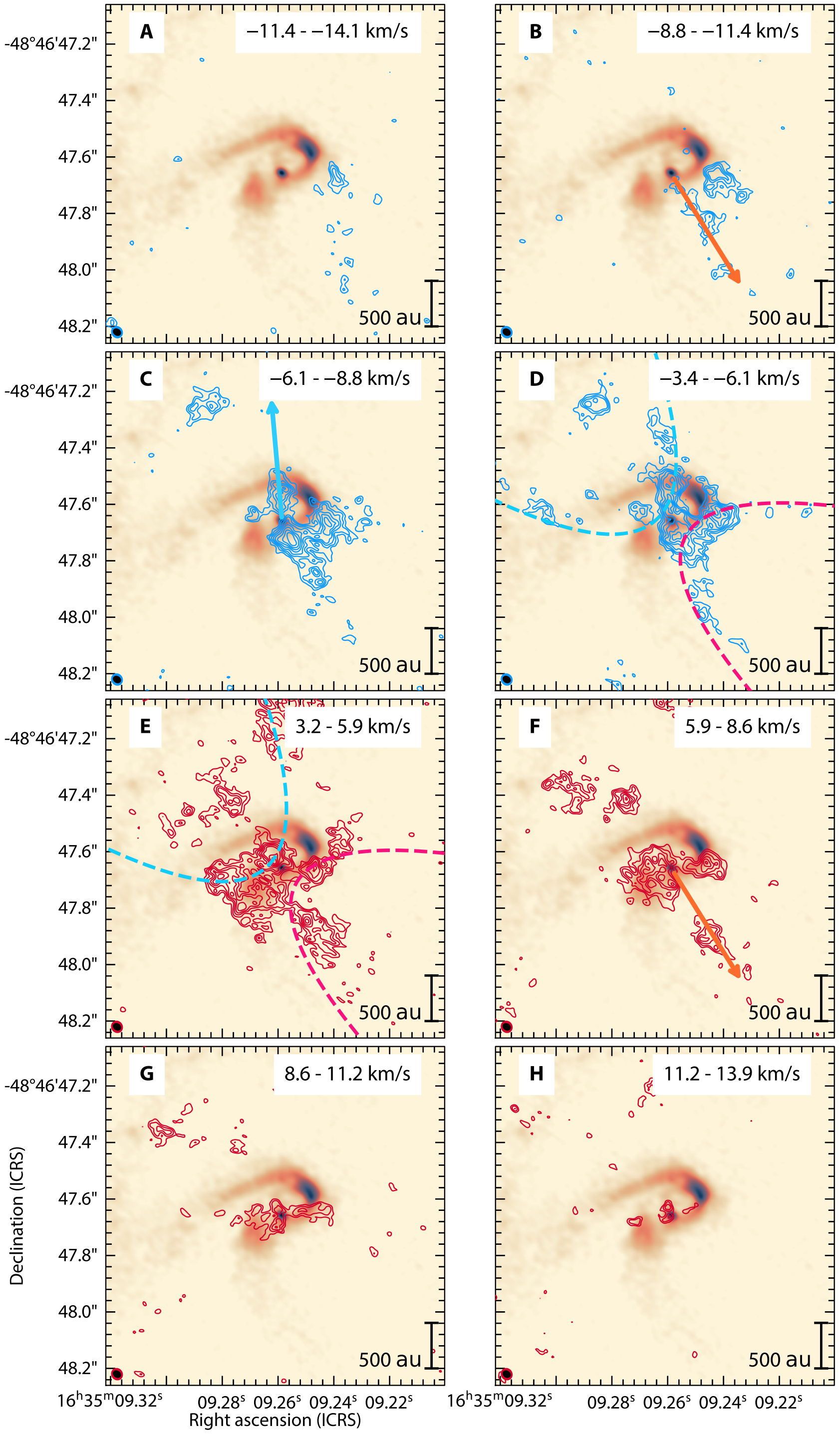

Figure S3: **SO $^3\Sigma$ v=0 J_K = $6_5 - 5_4$ channel averages in contours over 1.3 mm continuum.** The contour levels are 3, 4, 5, ... $\times\sigma$ with σ = 2.5 mJy beam^{-1} km s^{-1}. The blue and red dashed parabolas indicate the position and direction of the respective outflow cavities. The arrows indicate the direction of shocked gas likely tracing a jet component. The synthesized beams are shown in the bottom left corner for the continuum (black) and contours (respective color).

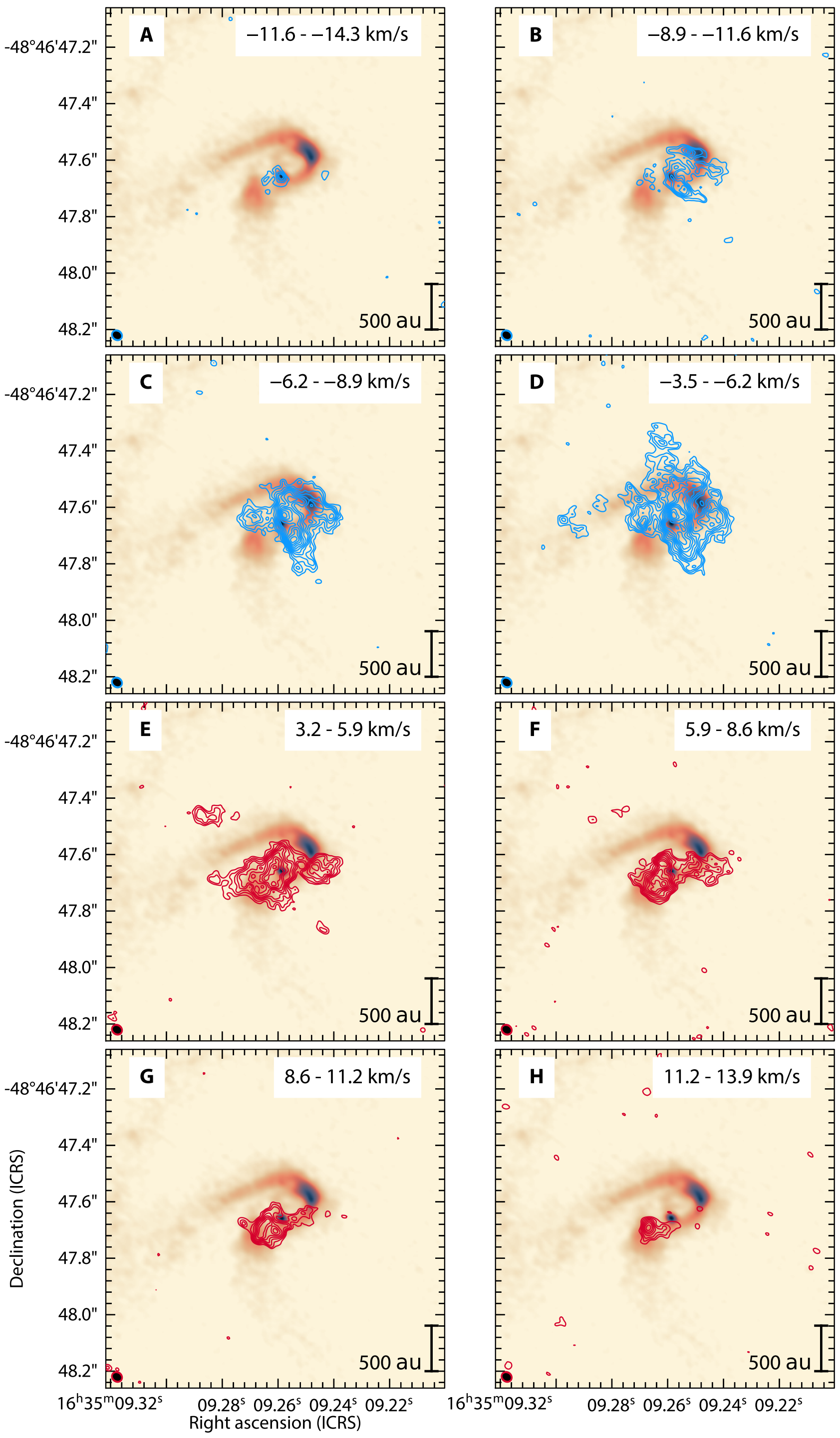

Figure S4: **SO $^3\Sigma$ v=1 $J_K = 6_5 - 5_4$ channel averages in contours over 1.3 mm continuum.** The contour levels are 3, 4, 5, ... $\times\sigma$ with $\sigma = 2.8\,\mathrm{mJy\,beam^{-1}\,km\,s^{-1}}$. The synthesized beams are shown in the bottom left corner for the continuum (black) and contours (respective color).
